# Computational biology and artificial intelligence in mRNA vaccine design for cancer immunotherapy

**DOI:** 10.3389/fcimb.2024.1501010

**Published:** 2025-01-20

**Authors:** Saber Imani, Xiaoyan Li, Keyi Chen, Mazaher Maghsoudloo, Parham Jabbarzadeh Kaboli, Mehrdad Hashemi, Saloomeh Khoushab, Xiaoping Li

**Affiliations:** ^1^ Shulan International Medical College, Zhejiang Shuren University, Hangzhou, Zhejiang, China; ^2^ Key Laboratory of Artificial Organs and Computational Medicine in Zhejiang Province, Shulan International Medical College, Zhejiang Shuren University, Hangzhou, Zhejiang, China; ^3^ Key Laboratory of Epigenetics and Oncology, the Research Center for Preclinical Medicine, Southwest Medical University, Luzhou, Sichuan, China; ^4^ Department of Biochemistry, Faculty of Medicine, Medical University of Warsaw, Warsaw, Poland; ^5^ Department of Genetics, Faculty of Advanced Science and Technology, Tehran Medical Sciences, Islamic Azad University, Tehran, Iran; ^6^ Farhikhtegan Medical Convergence sciences Research Center, Farhikhtegan Hospital Tehran Medical sciences, Islamic Azad University, Tehran, Iran

**Keywords:** neo-antigen mRNA vaccines, lipid nanoparticles, bioinformatics, artificial intelligence, targeted immunotherapy

## Abstract

Messenger RNA (mRNA) vaccines offer an adaptable and scalable platform for cancer immunotherapy, requiring optimal design to elicit a robust and targeted immune response. Recent advancements in bioinformatics and artificial intelligence (AI) have significantly enhanced the design, prediction, and optimization of mRNA vaccines. This paper reviews technologies that streamline mRNA vaccine development, from genomic sequencing to lipid nanoparticle (LNP) formulation. We discuss how accurate predictions of neoantigen structures guide the design of mRNA sequences that effectively target immune and cancer cells. Furthermore, we examine AI-driven approaches that optimize mRNA-LNP formulations, enhancing delivery and stability. These technological innovations not only improve vaccine design but also enhance pharmacokinetics and pharmacodynamics, offering promising avenues for personalized cancer immunotherapy.

## 1 Introduction

Messenger RNA (mRNA) vaccines have emerged as a rapid, flexible, and scalable strategy in cancer immunology. This innovative method elicits a robust and targeted immune response (Lorentzen et al., 2022; Yao et al., 2024). The effectiveness of mRNA vaccines during the COVID-19 pandemic has underscored their potential in addressing infectious diseases (Chakraborty et al., 2021; Pennisi et al., 2024). However, moving from concept to clinical implementation involves navigating significant scientific and technical challenges, necessitating a comprehensive, interdisciplinary approach (Lorentzen et al., 2022; Sayour et al., 2024). mRNA vaccines in oncology are considered personalized, representing a key advance in precision medicine by targeting the unique genetic mutations in an individual’s tumor cells (Lorentzen et al., 2022). By crafting a vaccine that targets these specific anomalies, this personalized method seeks to elicit a precise immune response, minimizing off-target effects and significantly enhancing therapeutic outcomes (May, 2024).

Unlike traditional vaccines, which use inactivated or attenuated pathogenic proteins, mRNA vaccines deliver tumor-associated antigens (TAAs) or neoantigens directly to antigen-presenting cells (APCs) like dendritic cells (DCs) or macrophages. After the tumor antigen is presented on the surface of the APCs, a cascade of immune responses is triggered, initiating adaptive immunity (Pardi et al., 2018; Gote et al., 2023). These neoantigens are processed and displayed on the cell surface via major histocompatibility complex (MHC) class I molecules, allowing the immune system to recognize the tumor proteins as foreign and triggering an immune response (Esprit et al., 2020). The primary immune response involves cytotoxic T lymphocytes (CTLs), which recognize and eliminate cancer cells expressing specific tumor antigens (Vishweshwaraiah and Dokholyan, 2022). Additionally, APC activation stimulates CD4+ T helper 1 (TH1) cells, which release cytokines to boost CTL activity and recruit macrophages, creating an immune-reactive tumor microenvironment (TME) (Li et al., 2022b; Ramirez et al., 2023). By enhancing the infiltration of immune cells, such as CTLs and macrophages, and overcoming immune checkpoint inhibition, mRNA vaccines can help shift the balance in favor of anti-tumor immunity. This reprogramming of the TME supports a more effective and sustained immune response against cancer cells, ultimately improving the overall efficacy of cancer immunotherapy (Gote et al., 2023; Ramirez et al., 2023).

In the case of naked mRNA vaccines, the mRNA is delivered directly into the body without any protective carrier. Once administered, the naked mRNA is taken up by cells, including DCs, through endocytosis or direct membrane fusion (Hasan et al., 2023). After entering the cytoplasm, the mRNA is translated into the target tumor antigen, which is processed and presented on MHC class I molecules, stimulating a robust immune response, specifically activating CTLs that target and destroy tumor cells expressing the same antigen. However, naked mRNA has some limitations, particularly in terms of stability and delivery efficiency (Abbasi et al., 2024).

To overcome these challenges, lipid nanoparticles (LNPs)-encapsulated mRNA are commonly used, and they are the only FDA-approved delivery vehicles for mRNA vaccines (Igyártó and Qin, 2024). LNPs are designed to encapsulate the mRNA, protecting it from degradation and improving its stability in the bloodstream. They also facilitate the efficient delivery of mRNA into target cells. Once inside the cell, the mRNA is released from the LNPs and enters the cytoplasm, where translation occurs, leading to the production of tumor antigens. LNPs are especially advantageous for improving cellular uptake. They interact with the cell membrane, facilitating endocytosis and ensuring that the mRNA is delivered into cells in a controlled manner. Once inside, the mRNA is translated into the antigen, processed, and presented by APCs on MHC class I molecules, leading to the activation of CTLs and the initiation of a strong anti-tumor immune response (Imani et al., 2024). By using LNPs, the delivery of mRNA vaccines becomes more efficient, enhancing both the stability of the mRNA and the ability of APCs to initiate a targeted immune response (Alameh et al., 2021; Shuptrine et al., 2024).

The development of personalized mRNA vaccines involves several crucial steps, each supported by advanced bioinformatics tools. Initially, next-generation sequencing (NGS) is used to analyze the genome of the pathogen or tumor, identifying unique mutations and neoantigens (Alburquerque-González et al., 2022; Al Fayez et al., 2023). Comprehensive genetic data is crucial for designing mRNA vaccines. Tools like NetMHCpan and the Immune Epitope Database (IEDB) identify the most immunogenic HLA-I and MHC class I epitopes to trigger a strong T-cell response (Kim et al., 2012; Cai et al., 2021). To enhance stability and efficiency, RNAfold and mfold predict the mRNA’s secondary structure, reducing degradation and improving effectiveness (Chen and Chan, 2023). LNP formulation tools, such as NanoAssembler, optimize delivery by protecting the mRNA and aiding its entry into host cells for effective antigen expression (Wang et al., 2022).

On the other hand, machine learning algorithms further refine these predictions by analyzing extensive immunological data. Incorporating machine learning and AI into this process is vital. Algorithms like Random Forest, Support Vector Machines (SVMs), and Convolutional Neural Networks (CNNs) analyze large datasets to predict vaccine efficacy and potential side effects. These AI-driven insights help optimize vaccine design, enhancing efficacy and safety (Bravi, 2024).

While advancements in bioinformatics and AI are significant, comprehensive comparative studies in this field are lacking, which limits our understanding of their full potential. This paper explores the role of these technologies in developing personalized mRNA vaccines, focusing on genome sequencing, epitope prediction, RNA structure analysis, and LNP formulation. We discuss the challenges, insights, and future directions, highlighting how AI improves vaccine development by analyzing data, identifying patterns, and optimizing design to predict side effects and enhance effectiveness. This paper aims to address current knowledge gaps and encourages further research in oncology and immunology, where personalized mRNA vaccines have the potential to transform cancer treatment.

## 2 Sequencing and initial data acquisition

**Figure 1:**
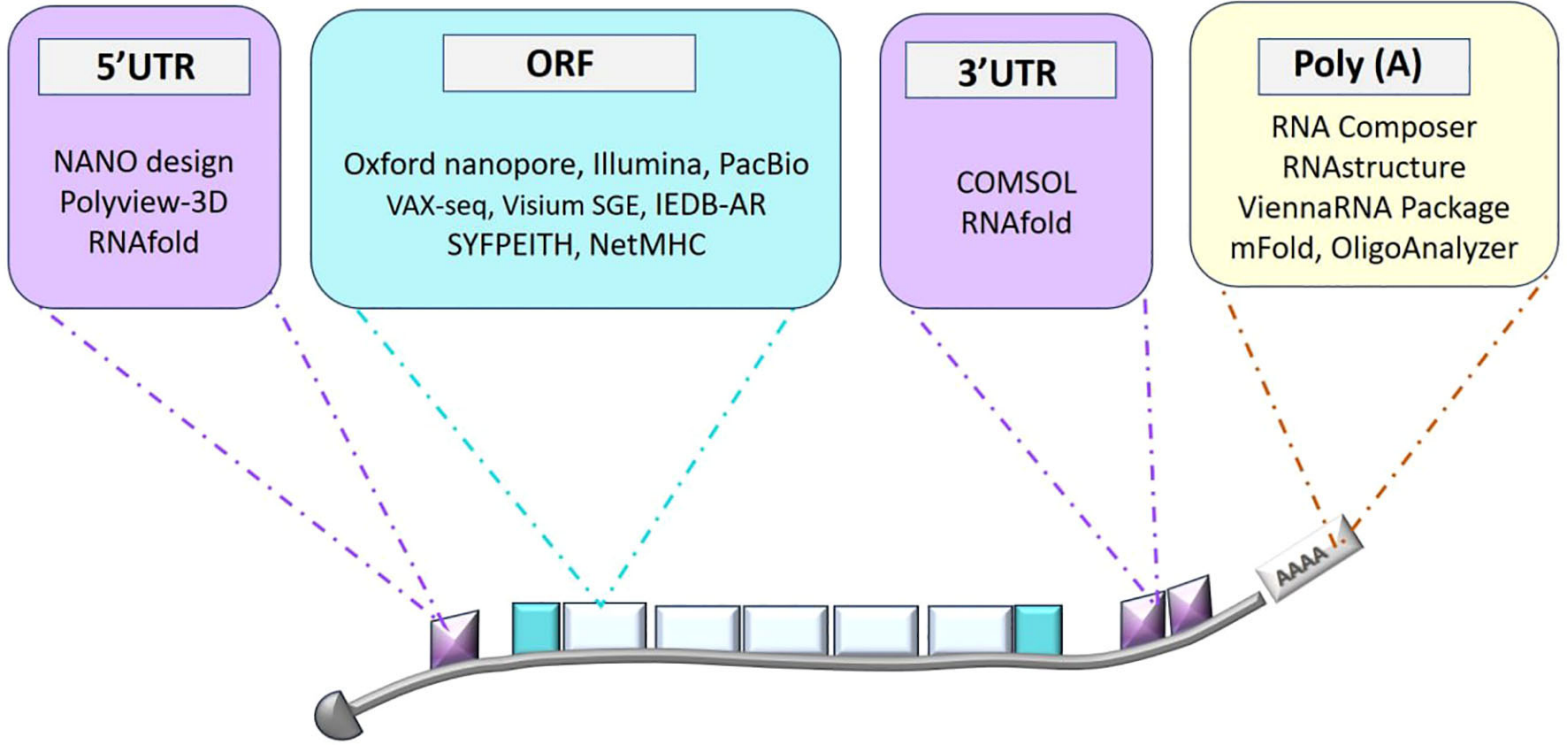
Overview of bioinformatics tools for mRNA structure prediction and design. This diagram highlights the various stages of mRNA design, including secondary structure prediction, coding sequence (CDS) optimization, and 3D structure modeling, along with the bioinformatics tools employed at each stage to enhance mRNA design for therapeutic applications.

**Figure 1** presents a schematic overview of bioinformatics tools for mRNA structure prediction and design, covering methods for secondary structure prediction, coding sequences (CDS) optimization, and 3D modeling. Sequencing and initial data acquisition are fundamental steps in developing mRNA vaccines, providing essential genetic information about target viruses, and setting the stage for vaccine design and optimization (Gunter et al., 2023). Key sequencing technologies such as Illumina, Oxford Nanopore, and PacBio play crucial roles in this process. Illumina’s high-throughput short-read sequencing offers extensive coverage of viral genomes, helping to identify genetic variations that are important for understanding viral diversity and evolution (Lemay et al., 2022). Oxford Nanopore’s real-time long-read sequencing provides insights into full-length RNA transcripts and complex genomic regions, which is useful for detecting diverse viral variants and structural features (Stefan et al., 2022). PacBio’s high-accuracy long-read sequencing allows for detailed genomic characterization and variant analysis, particularly beneficial for studying RNA viruses. Bioinformatics tools play a crucial role in maintaining the quality, preprocessing, and comprehensive analysis of sequencing data across all three sequencing technologies. FASTQC (Fast Quality Control) assesses key quality metrics like base quality scores and GC content, while Trimmomatic eliminates artifacts and adapter sequences from raw reads (Bolger et al., 2014), thereby improving the accuracy of subsequent analyses. SAMtools manages aligned sequences in Sequence Alignment/Map (SAM) and Binary Alignment/Map (BAM) formats, which is vital for variant calling and in-depth genomic analysis, offering valuable insights for vaccine design. The workflow begins with alignment tools such as the Burrows-Wheeler Aligner (BWA) and Bowtie, which align short-read mRNA sequences to reference genomes or transcriptomes (Rajan-Babu et al., 2021). Also, Visium Spatial Gene Expression (Visium SGE) is an advanced platform that combines spatially resolved transcriptomics with histological imaging to map gene expression within the structural context of tissues, enabling precise insights into cellular activity and tissue architecture (Toyama et al., 2023). These tools are instrumental in identifying conserved regions and potential immunogenic epitopes within the mRNA sequences. Following alignment, assembly algorithms reconstruct full-length mRNA sequences by integrating sequence overlaps and pairing information, ensuring the integrity and completeness of mRNA constructs for vaccine production.

In practice, during the development of mRNA vaccines for novel viral outbreaks, alignment tools like BWA are used to compare mRNA sequences with known sequences of related viruses. This process helps identify conserved regions critical for vaccine design, ensuring effective targeting of the virus and the induction of protective immune responses in vaccinated individuals. **Table 1** compares the main key sequencing technologies, including Illumina, Oxford Nanopore, and PacBio.

**Table 1:** Comparison of sequencing technologies for mRNA vaccine development.

Feature	Illumina	Oxford Nanopore	PacBio	VAX-seq	Visium SGE
Read length	Short-read	Long-read	Long-read	Short-read to long-read	Short-read to long-read
Tech.	Seq. by Fluor. nucleotides	Nanopore-based detect.	SMRT tech.	Sequencing of mRNA sequences	Spatial transcriptomics
Data	High-throughput	Real-time seq.	High-accuracy	High-throughput	High-throughput
Accuracy	Very high	Moderate	Very high	High	Moderate to high
Tools	BWA, FASTQC	Minimap2, GraphMap	HGAP, SMRT	Custom tools (specific to VAX-seq)	Space Ranger, Seurat
Complexity	Moderate	Lower	High	Moderate	Moderate
Seq. depth	Very high	Moderate	High	High	High
Turnaround	Moderate	Fast	Moderate	Fast	Moderate
Cost	Mod. to high	Moderate	High	Moderate	High
Capabilities	No	Yes	No	Yes	Yes
Suitability	High	High	Moderate	High	High
Advantages	Extensive coverage, high acc.	Real-time seq., long reads, min. lib. prep	High acc., long reads, detailed analysis	High-resolution immune profiling	Spatial resolution, gene expression mapping
Disadvantages	Short reads, complex lib. prep	Lower acc.	Higher cost, complex lib.	Limited to vaccine studies	Limited to tissue samples

Seq., Sequencing, Fluor., Fluorescent, Tech., Technology, Detect., Detection, SMRT, Single Molecule Real-Time, VAX-seq, Vaccination Sequencing, Acc., Accuracy, Min., Minimal, Lib., Library, Prep., Preparation, Genom., Genomic, Mod., Moderate, BWA, Burrows-Wheeler Aligner, BWT, Burrows-Wheeler Transform, FM-index, Full-text Minute-space Index, HGAP, Hierarchical Genome Assembly Process, FASTQC, FAST Quality Control, Trimmomatic, Trimming tool for quality control, Minimap2, Alignment tool for long reads, GraphMap, Alignment tool for long reads; SMRT Link; Software suite for managing SMRT sequencing data; Space Ranger, Software for analyzing Visium spatial gene expression data; Seurat, Tool for analyzing single-cell RNA-seq and spatial data.

### 2.1 Illumina

Illumina sequencing is a high-throughput technology known for its precision in generating short DNA or RNA sequence reads, which can produce fragments hundreds of bases in length and is vital for mRNA vaccine development. The process starts with fragmenting DNA samples of approximately 300-500 bp or RNA samples of about 200 bp, followed by the attachment of adapters. These fragments are then amplified on a flow cell through bridge amplification, forming clusters of identical sequences. During sequencing, fluorescently labeled nucleotides are incorporated into the growing DNA strands. Techniques like RELIC are used to correct dye bias in Illumina data, ensuring accurate sequencing results (Xu et al., 2017). Illumina can also help length-sequencing platforms such as ONT get high-quality genomes more efficiently (Lerminiaux et al., 2024). Each nucleotide emits a unique color when excited by a laser, and high-resolution cameras capture these colors to determine the nucleotide sequence. This technology is crucial for identifying genetic variations and viral genome features, aiding in the development of effective mRNA vaccines. For processing sequencing data, algorithms like BWA (Guo and Huo, 2024) and Bowtie are essential. BWA uses the Burrows-Wheeler Transform (BWT) (Keel and Snelling, 2018) for efficient sequence alignment, while Bowtie utilizes the FM-index for indexing and searching data (**Table 1**). Brittney N. Keel’s comparison shows that BWA is more robust, whereas HISAT2 is faster and uses less memory than both BWA and Bowtie2 (Keel and Snelling, 2018). Data quality is ensured with preprocessing tools such as FASTQC, which evaluates base quality scores, and Trimmomatic, which removes low-quality bases and adapter sequences to enhance alignment accuracy and variant detection. Detailed mathematical formulations and specific operational details of these methods are available in **Supplementary file S1**.

### 2.2 Oxford nanopore

Oxford Nanopore sequencing is a state-of-the-art long-read technology that plays a crucial role in mRNA vaccine development. This method uses nanopore sensors to detect changes in ionic current as nucleic acids pass through a protein nanopore (Xue et al., 2020), such that a negatively charged single-stranded DNA or RNA molecule is driven from the negatively charged “cis” side through the nanopore to the positively charged “trans” side, which is recorded and analyzed to infer the base sequence (Su et al., 2023). Currently, there are eight versions of the system, with R9 achieving an impressive translocation rate of 250 bases per second and R9.4 achieving a translocation rate of 450 bases per second, which is a significant improvement over R7’s 70 bases per second. The other different systems have their advantages (Wang et al., 2021).

For mRNA vaccine development, Oxford Nanopore sequencing has been modified to sequence them directly without reverse transcription. Although the accuracy of direct sequencing of RNA is lower than that of DNA sequencing, about 83% to 86%. Similarly, Oxford Nanopore sequencing also provides direct sequencing of complementary DNA (cDNA) without the need for polymerase chain reaction (PCR) amplification (Wang et al., 2021). This capability is essential for understanding the complete structure and function of RNA, including secondary structures and complex genomic regions vital for designing effective vaccines.

Key bioinformatics tools for Oxford Nanopore sequencing are MinION Knowledge Base (MinKNOW) and Guppy. MinKNOW manages the sequencing device and collects raw data (Oeck et al., 2023), while Guppy performs base-calling to convert the raw signal data into nucleotide sequences (Wick et al., 2019). After base-calling, alignment tools such as Minimap2 are used to map these long reads to reference genomes. During mRNA vaccine development, this technology allows real-time sequencing of viral genomes, aiding in the identification of conserved regions and potential epitopes crucial for effective vaccine design. Although the average accuracy of ONT sequencing is improving, certain subsets of reads or read fragments have very low accuracy, and the error-rate reads of 1D reads and 2D/1D reads are still much higher than the short reads produced by NGS technologies (Wang et al., 2021). Oxford Nanopore sequencing also excels at detecting viral variants by analyzing complete sequences and complex genomic regions, with the characteristics of short turnaround time and low cost (Xu et al., 2022). This ability to identify mutations and variations is essential for designing mRNA vaccines that elicit strong immune responses against diverse viral strains.

### 2.3 PacBio

PacBio sequencing, utilizing Single Molecule Real-Time (SMRT) targeting technology that does not require pausing between read steps, so kinetic changes interpreted from light-pulse movies can be analyzed to detect base modifications, such as methylation, and accurate detection and discovery of all variant types, even in hard-to-reach regions of the genome (Rhoads and Au, 2015), has the potential to revolutionize physical health, reproduction, cancer research, as well as microbial and viral genetic testing (Ardui et al., 2018), is crucial for mRNA vaccine development due to its capability to produce highly accurate long-read sequences. This technique involves DNA polymerase synthesizing complementary DNA strands with fluorescently labeled nucleotides. The emitted light from these nucleotides is detected in real-time, enabling immediate base calling. For RNA molecules, PacBio finds novel genes, transcripts, and alternative splicing through a complete view of transcript isoform diversity to sequence them (Rhoads and Au, 2015).

The long-read capability of PacBio sequencing, which can extend up to 60 kb, provides significant advantages in identifying and quantifying subtypes, including novel ones (Rhoads and Au, 2015). According to *Jia H.* et al. findings, this technology allows for low-input library preparation, requiring only 100 ng of DNA for the Sequel system and 400 ng for the Sequel II system (Jia et al., 2024). This is particularly useful for comprehensive viral genome sequencing, including the identification of new variations and genetic mutations in viruses like SARS-CoV-2 (Nicot et al., 2023).

The SMRT Link software suite manages data collection and processing, including base calling and error correction. Algorithms such as the Hierarchical Genome Assembly Process (HGAP) (Chin et al., 2013) and Canu et al. (Prjibelski et al., 2023) address the challenges of assembling long reads by correcting errors and constructing complete genome sequences. HGAP builds consensus sequences from long reads. PacBio sequencing is crucial for identifying conserved regions and potential immunogenic epitopes within viral genomes, which helps in designing effective mRNA vaccines. However, the technology has limitations, including lower throughput with fewer sub-reads or CCS reads and a higher error rate of about 11-15% for CLR reads (Rhoads and Au, 2015).

### 2.4 VAX-seq

VAX-seq, a novel sequencing technology, plays a pivotal role in advancing the field of mRNA vaccine development. This high-throughput sequencing method is specifically tailored for the identification and quantification of vaccine-induced immune responses (Gunter et al., 2023b). VAX-seq is a specialized technology focused on sequencing mRNA in the context of immune profiling. Its ability to detect modified nucleosides is limited and primarily inferred through indirect analyses or complementary assays (Gunter et al., 2023). By providing a more detailed understanding of the interactions between mRNA vaccines and the immune system, VAX-seq enables the identification of specific mRNA sequences that contribute to optimal immune activation. This technology allows researchers to profile the genetic composition of mRNA vaccines and their translation products with greater accuracy, improving both the design and efficacy of these vaccines (Gote et al., 2023).

One of the key advantages of VAX-seq over traditional sequencing methods, such as Illumina and Oxford Nanopore, lies in its ability to offer higher-resolution insights into the transcriptome (Gunter et al., 2023). This enables a more comprehensive analysis of vaccine-induced responses, allowing for the detection of rare or subtle immune reactions that might be missed with other methods. The technique enhances the ability to tailor mRNA vaccine sequences to better stimulate desired immune responses, which is crucial for optimizing vaccine formulations for various pathogens, including those that require more precise immune targeting. Incorporating VAX-seq into mRNA vaccine development holds significant potential for both enhancing vaccine design and guiding clinical decision-making. By combining its high sensitivity with the ability to sequence and quantify complex mRNA sequences, VAX-seq aids in the identification of critical sequence motifs and epitopes (Jeeva et al., 2021). This level of detail is essential for the development of more effective mRNA vaccines, capable of eliciting stronger, more targeted immune response, and ultimately providing better protection against infectious diseases (Gunter et al., 2023).

### 2.5 Visium SGE

Visium SGE by 10x Genomics has emerged as a transformative analytical tool, integrating spatially resolved transcriptomic data with high-resolution tissue histology (Ståhl et al., 2016). This platform allows researchers to map gene expression patterns directly onto histological sections, providing unparalleled insights into the spatial context of mRNA translation and immune cell dynamics within tissues (Toyama et al., 2023). Visium SGE combines spatial transcriptomics with high-throughput short-read sequencing. While it offers spatial resolution and gene expression mapping, its capability to detect modified nucleosides is restricted to indirect bioinformatic inferences (Williams et al., 2022). By combining transcriptomics with histopathological features, Visium enables the identification of specific cell populations and their molecular activities about their precise tissue location. For example, in the context of mRNA vaccine development, Visium can localize mRNA-encoded antigen expression to immune-competent regions, such as lymphoid aggregates, while simultaneously identifying structural changes in surrounding tissue architecture. This dual-layer information is invaluable for validating predictive models like AlphaFold, ensuring that computationally predicted antigens are accurately expressed and situated in biologically relevant microenvironments (Smith et al., 2024). In mRNA vaccine development, Visium has proven instrumental in refining antigen design and delivery strategies. For instance, studies leveraging Visium have demonstrated its capability to map DCs activity in lymphoid tissues following mRNA-LNP administration, directly linking antigen presentation to CTLs recruitment. In one example, Visium analysis identified specific tissue regions where mRNA vaccines encoding TAAs were translated most efficiently, allowing researchers to pinpoint the spatial co-localization of antigen-expressing cells and CD8+ T-cell activation zones. This spatial information guided the optimization of LNP formulations to ensure antigen delivery to DCs located in lymphoid-rich areas, thereby enhancing CTL priming and overall vaccine efficacy (Melo Ferreira et al., 2021; Hudson and Sudmeier, 2022).

Moreover, Visium facilitates the identification of off-target effects and unintended mRNA expression in non-target tissues, a critical consideration in vaccine safety profiling. For example, spatial transcriptomic analysis using Visium uncovered ectopic expression of mRNA constructs in hepatocytes during preclinical studies, revealing suboptimal LNP biodistribution. Based on these findings, LNP formulations were redesigned to incorporate specific targeting ligands that preferentially deliver mRNA to DCs while minimizing liver uptake (Ståhl et al., 2016). This iterative approach underscores the power of Visium in bridging computational predictions with experimental outcomes, ensuring the spatial fidelity of mRNA expression, and advancing the rational design of mRNA vaccines for cancer immunotherapy (Toyama et al., 2023).

## 3 Antigen and epitope prediction

Antigen prediction uses bioinformatics to analyze pathogen genomes or proteomes, identifying specific epitopes that trigger immune responses through various MHC classes or DC and macrophages (Capelli et al., 2023). In mRNA vaccine development, choosing the right antigen targets is essential for effective expression and a strong immune response. Neo-antigen prediction technologies enhance vaccine safety and effectiveness by finding highly immunogenic epitopes, which can shorten development timelines and reduce costs (Soria-Guerra et al., 2015). The following section will detail the specific tools used in this process. Selecting optimal epitopes is crucial for robust immune stimulation, and epitope prediction tools are key in developing effective mRNA vaccines.

### 3.1 NetMHC

NetMHC is a user-friendly bioinformatics tool that utilizes information from both data types for training on binding affinity and eluting ligand data, thus being used to predict peptide-MHC interactions, addressing the challenge of identifying peptides that effectively bind to MHC molecules. NetMHC has undergone several transformative updates since its inception in the early 2000s, embracing the latest computational advancements and significantly enhancing its database of interactions between peptides and MHC. These updates have incorporated sophisticated scoring matrices, intricate hidden Markov models, and cutting-edge artificial neural networks (ANNs) (Zhou et al., 2023), collectively enhancing the tool’s predictive capabilities and broadening its application scope within the field, for example, NetMHCpan-4.0 achieves better performance, and ligands in all cases are predicted with very strong eluting ligand likelihood values (Jurtz et al., 2017). It has become an essential resource in immunoinformatics, crucial for understanding how peptide fragments derived from pathogens can activate CD8+ T cells and trigger immune responses, particularly neoantigens in cancer immunology (Wu et al., 2023).

Cytotoxic T cells play a central role in the pathogenesis and immunomodulation of malignancies, and the binding of peptides to MHC molecules is the most selective single step in the antigen presentation pathway. It has recently been shown that over 90% of naturally occurring MHC ligands are identified with 98% specificity (Nielsen and Andreatta, 2016). In vaccine development, NetMHC evaluates the binding affinity between peptides and MHC molecules, aiding researchers in selecting optimal peptides for vaccine inclusion to induce robust CD8+ T-cell responses. This capability enhances vaccine specificity and efficacy by focusing on peptides with the strongest interactions. During the intricate process of vaccine development, the versatile NetMHC tool harmoniously integrates with existing peptide-MHC data, leveraging computational simulations to accurately predict potential antigen epitopes – a pivotal step in vaccine design. This training approach integrates larger data content and can directly learn the length of each MHC molecule from the experimental binding data to present the optimal peptide (Andreatta and Nielsen, 2016). NetMHC provides highly accurate predictions due to its use of extensive training datasets and advanced modeling techniques like neural networks and position-specific scoring matrices (PSSMs). While it focuses on MHC class I molecules, its performance depends on the quality and breadth of peptide-MHC interaction data and may require substantial computational resources. Although NetMHC is excellent at predicting MHC interactions, it does not cover all aspects of antigen processing and presentation, such as class II MHC interactions. Despite these limitations, integrating NetMHC into the vaccine development process greatly improves the design of specific and effective vaccines.

### 3.2 IEDB Analysis Resource

The IEDB-AR (Immune Epitope Database Analysis Resource) is a crucial tool for designing mRNA vaccines against variable antigens, especially for virus-based vaccines such as those targeting influenza viruses, SARS-CoV-2, and HIV.I EDB-AR has T cell epitope prediction tools, B cell epitope prediction tools, and tools for the analysis of known epitope sequences or sequence groups. The IEDB-AR platform stands as an ideal choice for addressing diseases characterized by substantial antigenic variation or requiring a robust, multifaceted immune response. Its applicability extends to a wide spectrum of conditions, including those associated with infections, allergies, autoimmune disorders, and transplantations, where its capabilities are particularly well-suited to inform and guide therapeutic strategies (Vita et al., 2018).

Several new tools have been added to IEDB-AR. Among the T cell epitope prediction tools are TepiTool, MHC-NP, Immunogenicity, CD4EpiScore, and Deimmunization. These tools have their different functions, such as TepiTool, It can be used to predict naturally processed MHC class I and II ligands, deimmunization of therapeutic proteins, and prediction of T cell immunogenicity beyond MHC binding affinity (Dhanda et al., 2019). IEDB-AR also adds a new tool called LYRA (Automated Modeling of Lymphocyte Receptors), which allows for the simulation of 3D structures of B and T cell receptors (Klausen et al., 2015), allowing for the prediction of canonical structures per cycle, when necessary.

By utilizing algorithms such as ANNs and SVMs to predict both class I and II peptide-MHC binding affinities, T-cell and B-cell epitopes, and cross-reactive epitopes (Yan et al., 2024), IEDB-AR identifies optimal antigenic targets, such as pHLA-target Ags (Gerber et al., 2020), to stimulate both CD4^+^ and CD8^+^ T-cell responses, as well as antibody responses. However, its effectiveness depends on the quality and comprehensiveness of the peptide-MHC interaction data, which can impact prediction accuracy. This variability in data coverage may affect the tool’s precision.

### 3.3 SYFPEITHI

SYFPEITHI, a free bioinformatics tool from the late 1990s, predicts peptide-MHC interactions for MHC class I and II molecules. Its user-friendly interface and high accuracy help identify peptides that bind to specific MHC molecules and predict epitopes (Zhang et al., 2023a). The database includes peptide sequences (approximately 200 peptide motifs and 2000 peptide sequences), anchor position, MHC specificity, source protein, source organism, and publication references. The tool employs PSSMs as its primary algorithm to evaluate the binding affinity of peptides to MHC molecules, which can sequence the MHC-eluting peptides directly. The adopted scoring approach simplifies the identification of promising vaccine candidates by providing detailed binding scores and rankings. *Chao Shen* et al. findings show that this method effectively balances scoring and docking tasks, making the selection process both rigorous and efficient (Shen et al., 2023). But instead of synthesizing and testing dozens or even hundreds of peptides, SYFPEITHi prescreens a set of peptides and enables epitope prediction of the sequence, restriction elements, and their respective motifs of proteins or their genes., which aids in the design of effective vaccines (Rammensee et al., 1999). The accuracy of SYFPEITHI’s predictions depends on the quality and completeness of the peptide-MHC interaction data, with gaps potentially affecting reliability. SYFPEITHI does not account for critical aspects of antigen processing and presentation, such as peptide transport into the endoplasmic reticulum via TAP, proteasome trimming, or competition for MHC binding. These factors are essential for a full understanding of immune responses and peptide presentation (Larsen et al., 2005; Lee et al., 2024a).

## 4 Codon optimization

Before analyzing mRNA structure, it’s essential to focus on the Coding Sequence (CDS) and codon optimization. Codon optimization is crucial for improving CDS expression in a host organism. This process involves modifying codons to match the host’s preferred codon profile, which enhances gene expression efficiency and reduces costs (Hanson and Coller, 2018). Codon optimization takes into account factors such as codon usage bias, tRNA abundance, GC content, and RNA secondary structure. By carefully selecting codon combinations, researchers can improve protein expression, reduce mRNA degradation, and enhance stability. This also impacts protein folding, post-translational modifications, and immunogenicity (Zhang et al., 2023b). Software tools like GeneOptimizer and JCAT (Java Codon Adaptation Tool) help in this process by choosing the most efficient codons based on the host’s tRNA abundance and codon usage patterns. Here is a summary of their advantages and disadvantages.

### 4.1 GeneOptimizer

GeneOptimizer is a powerful tool for optimizing DNA sequences. It uses a sliding window method to adjust codon usage, GC content, and other factors to improve translation efficiency (Fu et al., 2020). It handles large gene sequences and manages key processes such as transcription, splicing, translation, and mRNA degradation. GeneOptimizer can complete gene optimization in minutes. Synthetic genes were designed by uploading sequences, selecting expression systems, specifying cloning vectors, and sequence details. At the same time, based on the data related to a given organism and the user’s sequence requirements, the DNA sequence that is most suitable for the user’s research requirements is generated. Researchers can use this tool to select optimal codon combinations for specific organisms, enhancing gene expression efficiency and scaling up protein production to meet experimental needs. Despite being a premium tool, GeneOptimizer empowers users with the autonomy to meticulously craft gene sequences, circumventing the necessity for DNA templates. It achieves this through the implementation of sophisticated codon optimization and sequence alignment algorithms, exemplified by its utilization of sliding windows for refining multiparameter DNA sequences and FOGSAA for executing swift, global sequence alignments (Chakraborty and Bandyopadhyay, 2013). Importantly, GeneOptimizer enhances mRNA stability and prolongs its half-life within cells through codon optimization (Schwanhäusser et al., 2011; Luo et al., 2023). Optimized mRNA sequences, with more favorable codons, related studies have shown that using GeneOptimizer at the same dose can significantly increase protein expression and produce more antigen proteins, leading to stronger immune responses and improved disease prevention. However, altering mRNA sequences with GeneOptimizer may have some unknown risks, such as potential interactions with other RNA and proteins within cells, which could lead to adverse reactions or reduced vaccine efficacy.

### 4.2 JCAT

The JCAT uses advanced algorithms, such as the Codon Adaptation Index and the Relative Codon Adaptation model, to enhance the production of heterologous proteins and there is no need to manually define highly expressed genes. Significantly, JCAT not only enhances gene sequence design but also safeguards against undesirable outcomes such as the emergence of restriction enzyme cleavage sites and Rho-independent transcription terminators. *Grote* et al.*’s* study underscores this capability, demonstrating how JCAT successfully adapted the codon usage of the *P. aeruginosa* exbD gene to that of *E. coli* while simultaneously evading the formation of identical restrictive sites, ensuring the stability of CDS. On the output, JCAT can be either a graph or a CAI (Codon Adaptation Index) value given by the pasted sequence and the newly adapted sequence. In addition, users can calculate CAI values by uploading gene sequences in FASTA format (Grote et al., 2005), which can help researchers quickly understand key biological information during mRNA vaccine development. JCAT is usually a codon optimization of a single gene in the laboratory. Therefore, experiments are comparing the original *Pseudomonas aeruginosa* DNA sequence with the DNA sequence optimized for Escherichia coli to demonstrate the degree of optimization. JCAT is user-friendly, offering high automation and precision, which allows researchers to efficiently analyze and adjust codon combinations. This optimization improves mRNA vaccine expression levels in host cells and avoids Rho-independent transcription terminators in codon-optimized DNA sequences (Postle and Good, 1985; Ermolaeva et al., 2000).

JCAT is built on biological insights into translational optimization, particularly the significance of codon adaptation in heterologous protein production. By leveraging algorithms like the Codon Adaptation Index (CAI), it aligns codon usage with host-specific tRNA pools, improving translation efficiency and reducing translational errors (Sample et al., 2019). Studies have shown that codon optimization not only enhances protein yield but also stabilizes mRNA expression by avoiding undesired sequence features, such as Rho-independent transcription terminators, which can destabilize transcripts (Leppek et al., 2022). Biologically, JCAT addresses critical factors in mRNA vaccine development, such as ensuring optimal ribosome loading to maximize protein translation while avoiding ribosome clustering that could lead to mRNA degradation. The tool’s ability to safeguard against restriction enzyme cleavage sites and transcriptional terminators highlights its utility in designing sequences for experimental and therapeutic applications. These features align with the broader understanding of how codon adaptation influences mRNA stability and protein expression, making JCAT an invaluable resource for precise, biologically informed sequence optimization (Grote et al., 2005).

## 5 Secondary structure prediction

Predicting the secondary structures of mRNA, including elements like α-helices and β-sheets, is essential for understanding its tertiary structure and function (Jiang et al., 2023). This prediction helps identify regions prone to degradation, allowing researchers to optimize gene sequences for greater mRNA stability. By analyzing the secondary structure, scientists can design mRNA sequences that are more efficient for translation, thereby improving vaccine expression in the host. Additionally, understanding the mRNA structure aids in selecting the most effective delivery systems, ensuring that mRNA efficiently enters cells and translates into target proteins, which enhances vaccine efficacy. Notably, CRISPR-Cas gene editing technology exemplifies its immense potential in addressing disease-causing mutations stemming from various cellular origins, highlighting the transformative impact of such evaluations on biomedical research and therapeutics (Cheng et al., 2020), and guiding the selection of those that can elicit stronger immune responses. In this section, we compare key tools for predicting mRNA secondary structure: RNAfold, mFold, and Inverse Prediction of RNA Knot (IPKnot).

### 5.1 RNAfold

RNAfold, part of the Vienna RNA Package, uses a thermodynamic model, such as the nearest neighbor thermodynamic model (Calonaci et al., 2020) to predict RNA secondary structures by computing the minimum free energy (MFE) and the thermodynamic regularized RNAfold can be used to calculate folding fractions that are highly correlated with the true free energy (Sato et al., 2021). RNAfold predicts RNA secondary structures by analyzing sequence inputs along with folding constraints, algorithms, and energy parameters. Users can select options for dangling ends, modified bases, and SHAPE reactivity data. The output can be customized to include interactive RNA secondary structure maps, reliability annotations, or mountain plots.

While RNAfold does not engage in direct codon optimization, its profound capability in predicting mRNA structures lays a solid foundation for subsequent codon optimization endeavors. Notably, key regions within mRNA, such as the 5’ UTR, 3’ UTR, and Poly(A) tail, play pivotal roles in facilitating vaccine translation, where the application of advanced techniques like sparsification can further enhance their efficacy (Gray et al., 2024), and RNAfold can assist in optimizing these regions to enhance vaccine expression levels. RNAfold also has several servers, such as RNAalifold, which can predict a set of common structures of aligned DNA or RNA sequences (Hofacker et al., 2002), which can calculate the hybridization energy and base pairing pattern of two RNA sequences (Bernhart et al., 2006). However, accurate RNA sequence data is essential for RNAfold’s predictions, and due to the complex diversity of RNA sequences, there may be some margin of error. Analyzing longer mRNA sequences also requires more computational time, which can significantly extend the development cycle.

### 5.2 Mfold

**Figure 2:**
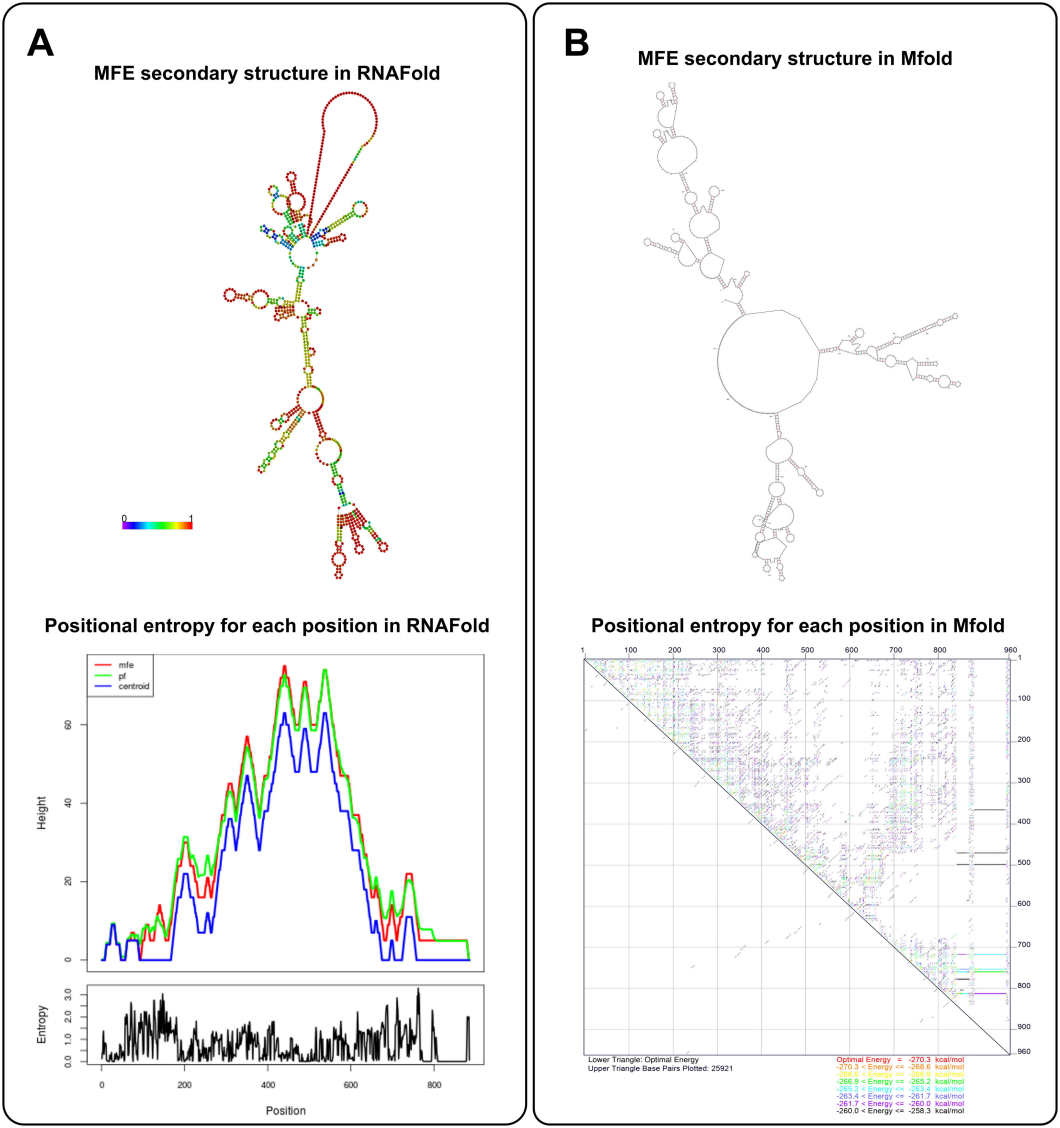
mRNA secondary structure prediction by RNAfold and Mfold. **(A)** The MFE structures for the template mRNA predicted by RNAFold and Mfold are shown. RNAFold (left) provides a detailed structural prediction with base-pairing probabilities, emphasizing stable regions-particularly in the UTRs and near the poly-A tail. This helps assess folding stability, essential for efficient translation. Mfold (right), while simpler, offers faster predictions, making it useful for quick structural overviews. **(B)** RNAFold’s positional entropy analysis shows low entropy in the UTRs and poly-A tail, confirming greater structural stability in these regions. Mfold provides a broader structural view but lacks detailed entropy data, making RNAFold more precise for stability assessment, while Mfold excels in speed and simplicity. The template used is a 962 bp mRNA encoding 12 neoantigens, with key regulatory elements like the HGH 5′ UTR, AES- mtRNA1-3′ UTR, and a 121-base pair long poly-A tail, designed for stability and efficient translation. The predictions were generated using RNAFold (ViennaRNA Package 2.4.18) and Mfold (version 3.6) (Zuker and Stiegler, 1981), with both tools sourced from their respective official repositories.

Mfold is a bioinformatics tool for predicting the secondary structure of RNA and DNA, similar to RNAfold. Similarly, Mfold contains several separate applications that can be used to predict nucleic acid folding, hybridization, and melting temperatures (Zuker, 2003). Mfold predicts the most likely secondary structure of a nucleic acid sequence by computing the most thermodynamically stable configuration (Zuker and Stiegler, 1981). This process involves calculating the free energy of various possible structures to determine which one is the most stable, which is essential for ensuring the stability of mRNA vaccines. Mfold uses dynamic programming algorithms to provide an optimal secondary structure based on the sequence and environmental conditions, such as Pknots-RE, NUPACK, gfold, and Knotty (Marchand et al., 2023) and the user can also change the rotation Angle to get the desired molecular folding orientation (Zuker, 2003). Unlike RNAfold, Mfold can identify regions in mRNA that might be prone to instability (Binet et al., 2023), such as regions with a high likelihood of forming secondary structures that may lead to degradation or poor folding. This sophisticated functionality empowers researchers to refine mRNA sequences with heightened precision for vaccine design, ensuring optimal performance. Furthermore, Mfold’s unique capability to anticipate the intricate interplay between mRNA molecules and delivery systems, and to visualize these interactions in various graphic formats including PostScript, PNG, or JPG, further augments its value in the realm of vaccine development (Zuker, 2003). **Figure 2** presents a detailed comparison between RNAFold and Mfold predictions for mRNA secondary structure. As shown in **Figure 2A**, RNAFold provides a comprehensive visualization, with a color-coded structure based on base-pairing probabilities. Warmer colors highlight highly stable regions, particularly within the UTRs and near the poly-A tail—key areas for mRNA stability and translational efficiency. This allows for an in-depth understanding of structural stability across the mRNA sequence. In contrast, Mfold offers a simpler structural model without color-coding or probabilistic information. While it generates quicker results, Mfold’s predictions lack the depth required for a thorough stability analysis.

RNAFold further enhances its predictions with a detailed entropy analysis, using overlapping curves for MFE, probable folding pathways, and Centroid structures to illustrate structural variability at each nucleotide (**Figure 2B**). This analysis confirms low entropy in regions like the UTRs and poly-A tail, indicating stability in these essential areas. Although Mfold provides an energy-based prediction, it does not offer the same clarity in entropy distribution. As a result, RNAFold’s combination of structural and stability data makes it better suited for precise applications, while Mfold remains useful for rapid, less detailed evaluations.

### 5.3 IPKnot

IPKnot is a specialized computational tool used to predict the secondary structure of mRNA molecules, providing critical insights into the folding process based on dynamic programming and thermodynamic principles (Kato et al., 2012). By simulating base-pairing interactions, IPKnot predicts structures such as hairpins, loops, and stems, which play a significant role in mRNA stability, translation initiation, and susceptibility to degradation by ribonucleases (Sato et al., 2011).

In the context of mRNA-LNP delivery, IPKnot’s folding predictions are essential for optimizing the interaction between mRNA and LNPs. The predicted mRNA secondary structure influences the mRNA’s ability to be encapsulated into LNPs, as well as the subsequent release and translation inside the target cell. IPKnot aids in designing mRNA sequences with secondary structures that are compatible with LNP formulations, enhancing encapsulation efficiency and promoting stable, controlled release into the cytoplasm. This stability is vital for maintaining the functional integrity of mRNA once inside the cell, ensuring that it can be efficiently translated to produce the encoded protein (Jabbari and Condon, 2014).

In cancer immunotherapy, specifically mRNA-based cancer vaccines, IPKnot plays a pivotal role in optimizing the mRNA sequence and its secondary structure for enhanced immune system activation. The folding pattern of the mRNA influences the conformation of the encoded antigen in MHC (Solheim et al., 1995). Efficient MHC class I and class II presentation is critical for triggering both CD8+ cytotoxic T cell responses and B cell-mediated antibody production against tumor-associated antigens. By fine-tuning the mRNA sequence to achieve an optimal secondary structure, IPKnot contributes to more efficient antigen presentation, thereby improving the activation of both the innate and adaptive immune systems. This leads to stronger and more sustained immune responses, which are essential for targeting and eradicating tumor cells (Bell et al., 2017). Moreover, IPKnot’s role in optimizing mRNA folding extends to improving the translational efficiency of mRNA in clinical applications, including gene therapies and personalized vaccines. The tool is integral in ensuring that mRNA molecules remain stable during synthesis, storage, and delivery, providing a foundation for the development of mRNA-based therapies with high efficacy and minimal degradation (Lee et al., 2024b). This capability is particularly crucial in the design of mRNA vaccines, where the accurate prediction of secondary structures ensures that the mRNA sequences are robust and capable of eliciting the desired immune response (Bon et al., 2008).

## 6 Protein structure prediction

**Figure 3:**
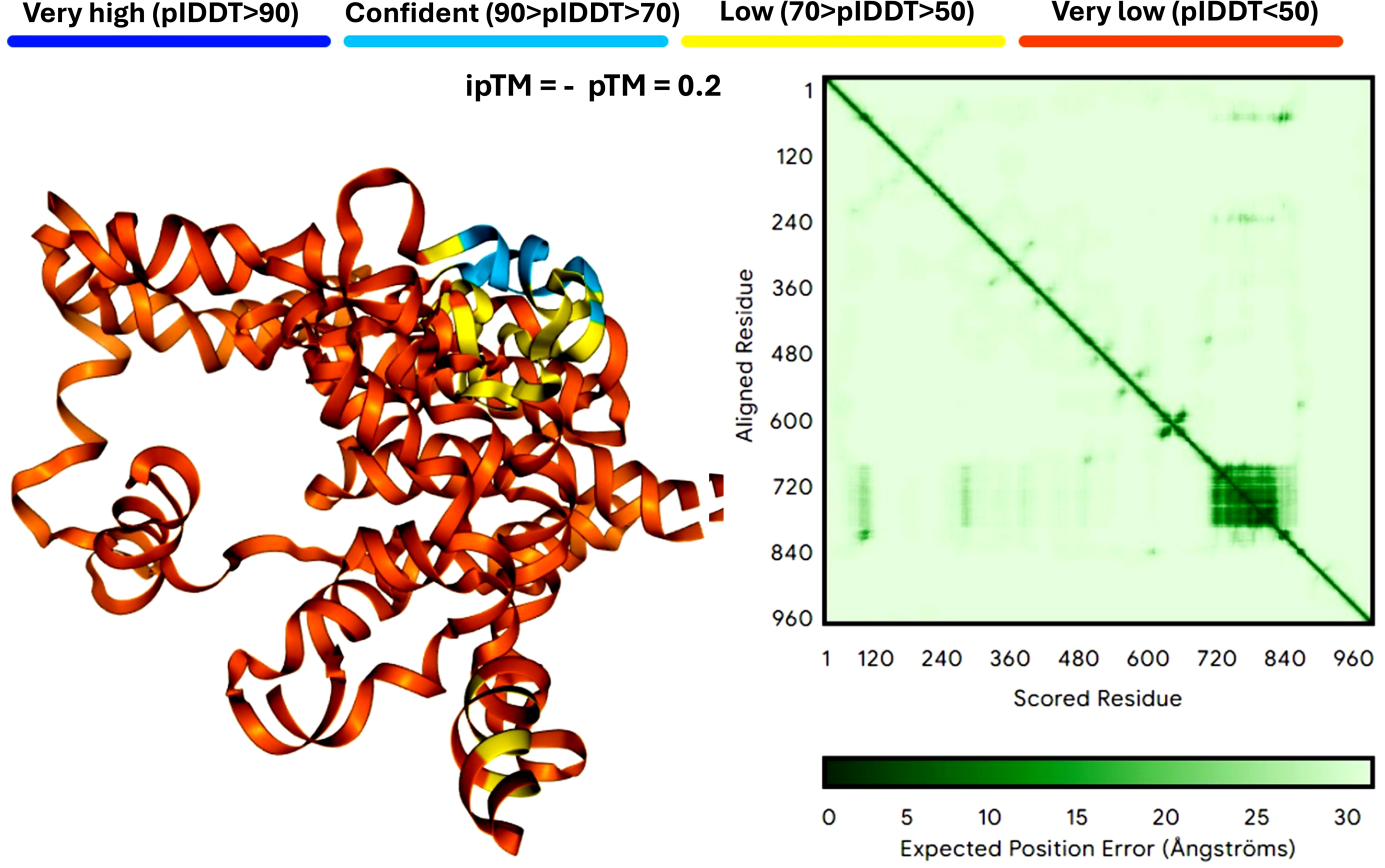
Comparison of mRNA structure predictions by AlphaFold. This figure illustrates the predicted three-dimensional structures of proteins derived from mRNA sequences, showcasing the strengths and limitations of AlphaFold. Panel A demonstrates AlphaFold’s capacity for detailed and accurate predictions for small to medium-sized proteins, while also highlighting its versatility in accommodating larger and more complex structures. The analysis is based on twelve neoantigens, featuring a YYA linker designed to enhance stability and facilitate efficient translation. The figure is powered by the latest version of AlphaFold 3 (accessible at https://alphafoldserver.com/) (Abramson et al., 2024). AlphaFold 3 is a web service that generates highly accurate biomolecular structure predictions for proteins, DNA, RNA, ligands, ions, and models chemical modifications for proteins and nucleic acids.

Protein structure prediction is crucial for understanding how mRNA vaccines generate their target antigens and interact within the host. Unlike costly proteomics techniques like gas chromatography-mass spectrometry (GC-MS), which analyze chemical compounds but don’t directly reveal protein structures, protein structure prediction provides theoretical insights crucial for refining antigen design before empirical testing. Accurate predictions ensure proteins fold correctly and function as intended, enhancing immune response. Key methods in this field include AlphaFold and Rosetta (Genc and McGuffin, 2025), which help identify potential folding and stability issues early, guiding experimental strategies and reducing extensive laboratory testing. **Figure 3** compares mRNA structure predictions from AlphaFold and Rosetta.

Protein structure prediction is essential for understanding how mRNA vaccines produce their intended antigens and how these proteins interact within the host. Distinct from intricate and expensive proteomics approaches, such as GC-MS, which delve into chemical compounds yet fall short in directly illuminating protein structures, protein structure prediction stands as a theoretical cornerstone for refining antigen design before empirical validation. Its precision is paramount, as it ensures that synthesized proteins adopt their correct folds and execute their intended functions, thereby fostering a potent immune response. Key methods, such as AlphaFold and Rosetta (Genc and McGuffin, 2025), are commonly used in this field. These approaches help identify potential issues in protein folding and stability early in the development process, guiding more effective experimental strategies and reducing the need for extensive laboratory testing.

### 6.1 AlphaFold

AlphaFold leverages deep learning techniques, such as the Attention Mechanism and Evolutionary Coupling algorithms, to predict the intricate 3D architectures of proteins from their amino acid sequences with atomic-level precision, even in the absence of prior structural knowledge (Jumper et al., 2021). **Figure 3** shows the results of mRNA structure predictions by AlphaFold. In particular, AlphaFold can handle the missing physical environment and generate accurate models in challenging situations, such as intertwined homologs or proteins that fold only in the presence of an unknown heme group (Abramson et al., 2024). At the same time, AlphaFold has greatly improved the accuracy of structure prediction by combining a novel neural network architecture and training program based on evolutionary, physical, and geometric constraints of protein structure (Jumper et al., 2021). It combines a novel neural network architecture and training program rooted in evolutionary, physical, and geometric constraints to achieve unparalleled accuracy. With a database exceeding 214 million predicted protein structures, AlphaFold has transformed structural biology and set new benchmarks in protein structure prediction (Varadi et al., 2022; Abramson et al., 2024; Varadi et al., 2024), which are crucial for designing vaccines. However, it demands significant computational power and is dependent on the quality of input data. It works well for small to medium-sized proteins but may struggle with very large or complex proteins due to these resource limitations. DeepMind’s AlphaFold has set a new benchmark in protein structure prediction, employing advanced deep learning frameworks such as CNNs and attention mechanisms to achieve unparalleled precision. With a database containing over 214 million predicted protein structures, AlphaFold has profoundly influenced structural biology, providing a median backbone accuracy of 0.96 Å r.m.s.d.95 and an all-atom accuracy of 1.5 Å r.m.s.d.95 (Jumper et al., 2021).

In mRNA vaccine development, AlphaFold’s detailed structural insights are pivotal for optimizing antigen design, ensuring their stability and immunogenicity. Its ability to model viral proteins encoded by mRNA is crucial for assessing antigenicity, which is essential for effective vaccine formulations (Asediya et al., 2024). Furthermore, AlphaFold plays a significant role in enhancing the development of mRNA-LNPs delivery systems (2020;Asediya et al., 2024). LNPs encapsulate and protect mRNA during systemic circulation, facilitating targeted delivery and efficient intracellular release. AlphaFold’s structural predictions can guide the design of mRNA sequences with stable secondary structures, such as stem-loops or pseudoknots, to improve binding affinity and stability within LNPs. This optimization enhances protection against nuclease degradation and ensures efficient delivery to target cells (2020).

Additionally, AlphaFold can refine mRNA designs to optimize release kinetics from LNPs within target cells. By predicting how mRNA structures interact with LNP components in response to intracellular conditions, such as pH or enzymatic activity, AlphaFold aids in developing formulations that promote efficient unpacking and robust antigen translation upon endosomal escape. These advances enhance DCs activation and antigen presentation to T cells, ensuring a potent adaptive immune response (Jumper et al., 2021; Olawade et al., 2024). AlphaFold also supports the design of mRNA elements encoding immune-stimulatory adjuvants, amplifying immunogenicity when combined with LNP formulations. By optimizing antigen stability and presentation by MHC molecules, AlphaFold contributes to tailored mRNA-LNP formulations that elicit durable and specific immune responses. This capability is particularly valuable in cancer immunotherapy, enabling more precise and effective vaccine designs (Oladipo et al., 2024).

### 6.2 Rosetta

Rosetta has become one of the leading computational tools for biomolecular structure prediction and design, using energy-based models, including Monte Carlo simulations and the Rosetta Energy Function (Rohl et al., 2004; Varadi et al., 2024), to predict 3D protein structures from mRNA sequences (Koehler Leman and Künze, 2023). It is useful for analyzing protein structures and interactions, and can also model antibodies and antigens (Schoeder et al., 2021), Rosetta can read most glycans in PDB files and automatically detect and score them, helping in the design of mRNA sequences to elicit strong immune responses. Rosetta provides detailed and adaptable predictions for proteins of various sizes, from small peptides to larger proteins, between 10-1000 residues (Du et al., 2021; Schmitz et al., 2021). However, it requires careful setup and parameter tuning, and its accuracy can decrease for very large or complex proteins.

## 7 Molecular dynamics simulations

MD simulations are crucial for understanding the intricate movements and interactions of atoms in mRNA molecules, especially how they interact with proteins and other cellular components (Hollingsworth and Dror, 2018). These simulations rely on several key parameters: Force fields are used to define atomic interactions, while temperature and pressure controls mimic physiological conditions. The permeability of lipid membranes is also considered to model interactions accurately (Venable et al., 2019). Time steps in simulations balance accuracy with computational efficiency. Cutoff distances manage non-bonded interactions, and periodic boundary conditions help minimize edge effects, enhancing the realism of the model. Solvent models simulate the surrounding aqueous environment, providing a more comprehensive view of mRNA behavior. Electrostatic treatments handle long-range interactions, ensuring that all aspects of the mRNA’s behavior are captured accurately. Simulation length and advanced techniques, such as replica exchange (Bock et al., 2023), offer deeper insights into mRNA dynamics. By optimizing these parameters, researchers can ensure that mRNA vaccines maintain their structure and function, thereby improving their effectiveness and immunogenicity. However, there are MD simulation tools for mRNA vaccine development (**Table 2**).

**Table 2:** MD simulation tools for mRNA vaccine development.

Feature/Tool	GROMACS	AMBER	NAMD	Rosetta	CHARMM
Primary Use	Large-scale sims	Struct. pred. & refine	Large-scale sims	Prot. model & design	Prot. model & design
Key Algorithms	PME, Parallel Proc.	GB model, PME	PME, MTS integr.	MC, Energy funcs	C36, Leapfrog integr.
Force Fields	Adv. for macro.	ff14SB, NA	CHARMM	Prot.-focused	C36, General
Strengths	Efficient, large sys.	Accurate, energy anal.	Large-scale, dyn. studies	Prot. design, interact.	mRNA-prot. anal.
Learning Curve	Mod.	Steep	Mod.	Mod.	Steep
Computational	High	High	Very high	Mod.	High
Advantages	Fast, ext. data integ.	Energy & struct. anal.	Large-scale, precise elect.	Prot. fold. & design	Free energy & struct. anal.

Sims, Simulations; Struct. pred. & refine, Structure prediction and refinement; Prot., Protein; Model, Modeling; MC, Monte Carlo; Funcs, Functions; Proc., Processing; Adv., Advanced; Macro., Macromolecules; NA, Nucleic Acids; Integr., Integrator; Dyn., Dynamic; Sys., Systems; Anal., Analysis; Interact., Interactions; Mod., Moderate; Elect., Electrostatics; Ext., External; Integ., Integration; Fold., Folding; C36, CHARMM36 (a force field).

### 7.1 GROMACS

GROMACS is a leading MD simulation tool known for its efficiency and precision. GROMACS can use MD, stochastic dynamics, or path integration methods to simulate any molecule in a solution or crystal, minimize molecular energy, analyze conformation, etc. Its simulation package includes GROMACS force fields (proteins, nucleotides, sugars, etc.) and can range from glass and liquid crystals to polymers, crystals, and biomolecular solutions. It effectively models the movements and interactions of atoms and molecules using advanced force fields (Rawat et al., 2021). GROMACS stands out as an exceptional tool for capturing the intricate dynamics and binding mechanisms of complex macromolecules, particularly mRNA-protein complexes. Its prowess lies in the employment of advanced algorithms, such as the Particle-Mesh Ewald (PME) method, which necessitates seamless all-to-all communication between the computational nodes (Kohnke et al., 2020). This sophisticated approach ensures that GROMACS can accurately model and analyze even the most challenging molecular interactions, providing unparalleled insights into their behavior, for accurate long-range electrostatics and supporting parallel processing for large-scale simulations. This makes it ideal for assessing the stability and behavior of biomolecular structures, crucial for optimizing vaccine designs. While GROMACS provides a powerful toolkit, beginners might need some time to learn how to use it, especially if integrating with R for data analysis and visualization, such as YAMACS, which can show the results in real time (Sarkar et al., 2022).

### 7.2 AMBER

Assisted Model Building with Energy Refinement (AMBER) is a well-known MD simulation tool used to predict and refine the 3D structures of mRNA. AMBER excels in offering intricate energy calculations and structural analyses of mRNA vaccines, thanks to its harnessing of efficient parallel computing and cutting-edge algorithms. The integration of the Generalized Born model and the Particle-Mesh Ewald method, among others, ensures that the simulations capture every nuance of the molecular interactions. AMBER has a variety of force fields suitable for different biomolecules, such as AMBER force field, CHARMM force field, etc., which can accurately describe the physical and chemical properties of various biomolecules. Furthermore, AMBER’s specialized force fields, notably the ff14SB and nucleic acid force fields, contribute to the highly accurate modeling of nucleic acids and proteins, providing unparalleled insights into the behavior of mRNA vaccines (Mikhailovskii et al., 2022). The package includes advanced techniques for energy minimization and refinement, such as the conjugate gradient and steepest descent methods, which ensure precise structural optimizations. Additionally, AMBER’s detailed analysis tools, including the Markov State Models and Principal Component Analysis, deliver deeper insights into tremolo-MD interactions of the mRNA vaccine. Its capabilities for implicit solvation and advanced free energy calculations not only study protein folding but also enhance the understanding of biomolecular stability and interactions (Shao and Zhu, 2018; Mikhailovskii et al., 2024).

### 7.3 NAMD

Nanoscale molecular dynamics (NAMD)’s advanced parallel computing techniques offer significant benefits for the development of neo-antigen mRNA vaccines. Its capability to perform large-scale simulations (Acun et al., 2018), involving millions of atoms, enables detailed modeling of neo-antigen mRNA vaccines. The use of sophisticated force fields, such as CHARMM, ensures accurate modeling of interactions between neo-antigen mRNA and proteins, which is crucial for predicting how neo-antigens are presented to immune cells and how they might stimulate an immune response. NAMD’s PME method stands as a testament to its precision in modeling long-range electrostatic interactions. This innovative approach enables the calculation of complete, non-truncated electrostatic interactions at a minimal computational cost, ensuring that the simulations are both accurate and efficient. With NAMD, researchers can gain unparalleled insights into the intricate electrostatic behavior of their molecular systems (Phillips et al., 2005, 2020). For neo-antigen mRNA vaccines, this means accurately simulating the electrostatic interactions between mRNA and protein targets, which is essential for understanding binding affinities and stability. Additionally, the Multiple Time-Step (MTS) integrator allows NAMD to handle different time scales efficiently (Phillips et al., 2005; Pechlaner et al., 2021), which is particularly useful for studying the dynamic behavior of neo-antigen mRNA and its interactions over time, offering insights into how these interactions evolve and affect the vaccine’s efficacy.

### 7.4 Rosetta

Rosetta is a versatile molecular modeling tool used primarily for protein structure prediction, protein-protein, protein-peptide complexes, and protein-ligand docking, and the design of biomolecules, can also model the RNA molecules in 3D structure (Koehler Leman and Künze, 2023). Unlike traditional MD simulation tools, Rosetta employs energy functions and Monte Carlo sampling methods to explore molecular interactions, and uses standard off-the-shelf computational hardware and all-atomic force fields to model the Large-scale conformational changes in proteins (Alford et al., 2017; Heilmann et al., 2020). Only a handful of structural biomolecule modeling frameworks have similar capabilities to Rosetta, covering applications of structural prediction and experimental data modeling, as well as protein design and small molecule drug discovery (Koehler Leman and Künze, 2023). It excels at predicting protein folding and designing new protein structures, making it valuable for integrating mRNA sequences with protein components to improve vaccine design. Rosetta’s ability to model protein interactions and design novel biomolecules complements MD simulations by providing additional insights into the structural and functional aspects of mRNA vaccines, and Some protein sampling limitations were overcome by the combination of MD simulation and Rosetta (Lindert et al., 2013).

### 7.5 CHARMM

CHARMM (Chemistry at Harvard Macromolecular Mechanics) is a sophisticated MD simulation package known for its in-depth analysis of biomolecular systems. It employs advanced force fields and simulation algorithms to model the movements and interactions of molecules over time (Brooks et al., 2009). CHARMM excels in the study of nucleic acids and proteins, thanks to its highly detailed force fields, such as CHARMM36 and the CHARMM General Force Field. These force fields allow for the precise modeling of mRNA structures and their interactions with proteins, which is essential for understanding the stability and behavior of mRNA vaccines. The software also offers robust integration methods, including the Verlet algorithm and the Leapfrog integrator, which significantly enhance the accuracy and efficiency of simulations. Moreover, CHARMM’s advanced energy minimization techniques, like the conjugate gradient and steepest descent methods, ensure thorough structural optimization of biomolecules (Jo et al., 2017). Additionally, CHARMM supports various analyses, such as free energy calculations, principal component analysis, and MD trajectory analysis, providing comprehensive insights into the dynamics and stability of mRNA and its interactions. In vaccine development, CHARMM’s capacity to simulate the intricate interactions between mRNA and protein components provides crucial insights into how these interactions impact vaccine efficacy. The package’s capabilities for modeling complex biomolecular systems and its extensive set of tools for analysis make it a powerful choice for researchers focused on optimizing mRNA vaccines and other biomolecular studies.

## 8 mRNA-LNPs formulation

**Figure 4:**
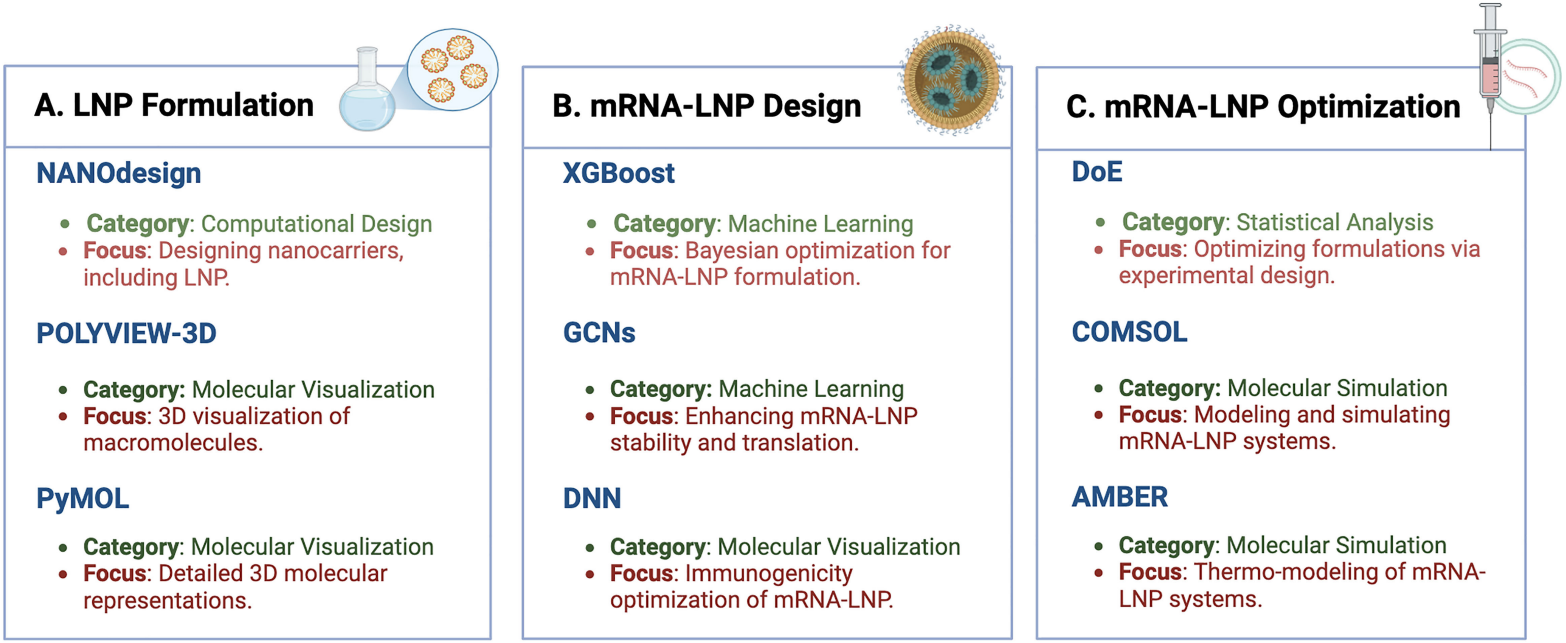
Integrated *in silico* framework for mRNA-LNP design, formulation, and optimization. This figure provides a comprehensive overview of the design and optimization process for mRNA-LNPs. **(A)** LNP formulation. This section highlights the tools used for nanoparticle formulation. NANOdesign enables computational modeling of nanocarriers like LNPs. POLYVIEW-3D and PyMOL offer molecular visualization for analyzing macromolecules and creating detailed 3D models of LNP structures **(B)** mRNA-LNP design. Advanced machine learning approaches, such as XGBoost are used for the optimization of mRNA-LNP formulations. Graph convolutional networks (GCNs) enhance mRNA stability and translation through better lipid and matrix design, while deep neural networks (DNNs) assist in optimizing immunogenicity. **(C)** mRNA-LNP optimization. DoE (Design of Experiments) employs statistical methods to streamline and improve formulations. COMSOL and AMBER simulate molecular and thermodynamic properties of LNPs to predict their behavior under various conditions, advancing their delivery efficiency. This figure was created using BioRender to incorporate high-quality symbols and illustrations for clarity.

LNPs are the only FDA-approved carriers for mRNA vaccines, ranging from 70 to 200 nm in size. They are crucial for encapsulating and stabilizing mRNA molecules, facilitating their effective delivery into target cells (Li et al., 2022a). The structural composition of LNPs, typically including lipids, cholesterol, and polyethylene glycol (PEG) (Hald Albertsen et al., 2022), directly influences their efficiency and efficacy. Key structural features, including lipid headgroup interactions and the arrangement of hydrophobic tails, play a critical role in the ability of LNPs to fuse with cell membranes and effectively deliver mRNA payloads. Designing and optimizing LNPs for mRNA cancer vaccines demands advanced computational tools capable of modeling and visualizing these complex structures and interactions. Tools such as NANOdesign, POLYVIEW-3D, and PyMOL are indispensable in this process. **Figure 4** depicts the various sections of the mRNA-LNP complex that must be designed, optimized, and characterized using these bioinformatics tools. This figure highlights how these tools contribute to achieving stable, functional, and highly efficient mRNA-LNP formulations, addressing aspects from pharmacology to pharmaceutical applications. By utilizing these resources, researchers can fine-tune parameters such as lipid composition, particle size, and surface properties to improve the performance and stability of LNPs in mRNA cancer vaccines.

### 8.1 mRNA-LNPs design tools

#### 8.1.1 NANOdesign

NANOdesign is a specialized tool for designing and optimizing LNPs for mRNA vaccines. Nanostructures can be formed by artificial design or by natural self-assembly mechanisms, which rely on intermolecular forces to automatically arrange into ordered structures. It provides detailed capabilities for modifying lipid types and ratios, essential for achieving optimal mRNA encapsulation and stability.

NANOdesign allows for comprehensive adjustments to lipid composition, which directly impacts the efficiency of mRNA encapsulation (Li et al., 2023). Researchers can explore different lipid types, such as phospholipids, ionizable lipids, and cholesterol, to determine the optimal combination for creating stable and effective nanoparticles. By adjusting these components, they can influence the fluidity and permeability of the lipid bilayer, directly impacting the retention and protection of mRNA within the LNP. NANOdesign also controls particle size, which is crucial for effective cellular uptake. It simulates how different formulation parameters impact the size and uniformity of the nanoparticles, ensuring they fall within the ideal range for delivery. Additionally, NANOdesign allows for modifications to surface properties, such as charge and hydrophilicity, which can alter cell membrane permeability. These adjustments can enhance the vaccine’s efficacy and improve its overall stability (Salatin et al., 2015). The tool models the release kinetics, encapsulation efficiency, and stability of mRNA within the LNPs, allowing researchers to optimize the release profile for controlled delivery. It also assesses the stability of LNPs under various conditions, including temperature and pH changes, to ensure the mRNA remains intact throughout storage and administration.

#### 8.1.2 POLYVIEW-3D

POLYVIEW-3D is an advanced tool designed to visualize and analyze the 3D structures of mRNA-LNPs, playing a crucial role in the design and optimization of these nanoparticles for mRNA delivery (Porollo and Meller, 2007). This software enables researchers to create highly detailed 3D models of LNPs, allowing for a deeper understanding of how these particles interact with mRNA and cellular components (Porollo et al., 2004). By providing clear, high-resolution representations of the nanoparticles, POLYVIEW-3D helps scientists examine the precise arrangement of lipids and other key components within LNPs, which is vital for optimizing their structure for effective mRNA encapsulation, stability, and delivery (Porollo and Meller, 2010).

One of the primary advantages of POLYVIEW-3D is its ability to model lipid bilayer formation and nanoparticle morphology. The software allows researchers to simulate how lipids are organized within the nanoparticle, which is essential for determining the overall stability and functionality of LNPs. Lipid composition plays a crucial role in the efficiency of mRNA encapsulation, as well as the nanoparticle’s ability to protect and deliver mRNA to target cells (Abumanhal-Masarweh et al., 2019). POLYVIEW-3D enables users to explore how variations in lipid composition—such as the inclusion of ionizable lipids, phospholipids, and cholesterol—affect the nanoparticle structure, influencing factors such as encapsulation efficiency, particle size, and surface charge. By adjusting these parameters, researchers can fine-tune the LNP design for optimal mRNA delivery (Watson et al., 2005; Arno et al., 2020).

The tool is especially valuable for examining LNP interactions with cell membranes. POLYVIEW-3D leverages the fluorescent marker Rhodamine 123 (Rho123) to visualize and analyze the activity of the P-glycoprotein membrane transporter in the MDCKII-MDR1 transgenic cell line (Sklenářová et al., 2021). This analysis is crucial for understanding how LNPs are taken up by cells, including immune cells like DCs, which play a pivotal role in the immune response to mRNA vaccines. By studying these interactions, POLYVIEW-3D aids in ensuring that mRNA-LNPs are efficiently delivered to the appropriate target cells, such as DCs, and interact with key receptors, including TLRs, to trigger the desired immune response (Porollo and Meller, 2007).

POLYVIEW-3D also facilitates the analysis of nanoparticle morphology and shape, which are key factors in the effectiveness of LNPs. Nanoparticle shape influences how LNPs interact with cell membranes during endocytosis and how they release their encapsulated mRNA into the cytoplasm (Mrazek et al., 2014). By visualizing and manipulating the structure of LNPs, researchers can assess how changes in shape and size impact the delivery process. This is particularly important for optimizing the stability and function of LNPs, as irregularities in shape or size may affect their ability to cross cellular membranes or release mRNA efficiently (Byrgazov et al., 2013).

Another important application of POLYVIEW-3D is in the design of nanocomposite hydrogels, which are often used in conjunction with LNPs to improve the stability and delivery of mRNA vaccines (Baigorria et al., 2023). The software allows researchers to model how changes in lipid composition or particle size affect the hydrogel’s properties, helping to optimize the gel’s characteristics for enhanced mRNA delivery (Ege et al., 2023). Nanocomposite hydrogels can provide additional protection for LNPs during storage and transport, and POLYVIEW-3D helps ensure that the LNPs remain stable and effective under different conditions (Baigorria et al., 2023).

By offering these capabilities, POLYVIEW-3D plays a pivotal role in the optimization of mRNA-LNP vaccines. Its ability to model complex interactions at the molecular level, visualize the structural components of LNPs, and analyze their interactions with cell membranes makes it an essential tool for researchers working to improve mRNA vaccine formulations. Whether fine-tuning lipid composition, modeling particle morphology, or studying cellular uptake, POLYVIEW-3D enables researchers to optimize LNP designs for maximum efficacy and stability in mRNA vaccine development (Bates et al., 2001; Porollo et al., 2004).

#### 8.1.3 PyMOL

PyMOL is a widely used molecular visualization and analysis tool that plays a critical role in designing and optimizing mRNA-LNP vaccines. This software enables researchers to create high-resolution, 3D representations of molecular structures, providing deep insights into the interactions between lipids, mRNA, and cellular membranes (Rigsby and Parker, 2016). The ability to visually manipulate and analyze the structures at the atomic level makes PyMOL an invaluable tool for optimizing LNP formulations (Mooers, 2020; Martí-Centelles et al., 2024).

PyMOL helps researchers build and visualize the three-dimensional structures of LNPs. By displaying how lipids are arranged within the nanoparticle, PyMOL allows for detailed structural analysis, including the packing of lipids in the bilayer (Wang and Deserno, 2010). This visualization aids in understanding how changes in lipid composition impact the overall stability and encapsulation efficiency of mRNA. By adjusting parameters like lipid chain length, headgroup types, and ionizable lipids, researchers can explore how these factors influence the structural integrity of LNPs and their ability to encapsulate mRNA efficiently.

PyMOL, using the molecular lipophilicity potential (MLP), a well-established method to calculate and visualize lipophilicity in molecules, allows researchers to observe interactions between the hydrophilic and hydrophobic regions of lipids and the charged and polar components of mRNA. By modeling these interactions, PyMOL helps identify optimal lipid compositions that improve mRNA encapsulation and stability, ensuring that the mRNA remains protected during delivery and is efficiently released once the LNP reaches its target cells (Oberhauser et al., 2014).

One of the critical steps in LNP design is the selection of the appropriate lipid mixture. PyMOL provides a platform to examine how varying lipid components—such as phospholipids, cholesterol, and ionizable lipids—affect the structural and functional properties of LNPs Seeliger and de Groot, 2010. By visualizing the changes in nanoparticle morphology and surface charge as lipid composition is modified, researchers can determine the best formulation for maximizing mRNA encapsulation, delivery efficiency, and stability under physiological conditions. PyMOL helps fine-tune these compositions, optimizing the LNP’s ability to deliver mRNA effectively while maintaining stability during storage and transport (El Khoury et al., 2023). In addition to lipid composition, the surface properties of LNPs, including charge, hydrophobicity, and hydrophilicity, play a crucial role in their interaction with cellular membranes. PyMOL allows researchers to model how altering these properties impacts the nanoparticle’s ability to be taken up by cells. PyMOL helps in understanding how LNPs interact with cellular membranes during the process of endocytosis. By simulating the insertion of LNPs into the lipid bilayer of a cell membrane, PyMOL enables researchers to visualize how LNPs may fuse with the membrane and release mRNA into the cytoplasm. This insight is vital for designing LNPs that optimize cellular uptake and ensure the efficient delivery of mRNA into cells for translation (Yong, 2015). PyMOL allows researchers to visualize the impact of particle size and morphology on the functionality of LNPs. Particle size is critical for effective cellular uptake, and by using PyMOL, researchers can simulate how varying nanoparticle sizes and shapes affect the overall performance of mRNA delivery. The tool helps visualize how the size and shape of the LNPs influence their stability, encapsulation efficiency, and release kinetics, which are essential factors for improving vaccine efficacy (Cao et al., 2020; Sebastiani et al., 2021).

PyMOL can also be used to simulate the effects of various environmental conditions such as pH, temperature, and ionic strength on the stability and function of LNPs. By visualizing how LNPs change under different conditions, researchers can predict the behavior of the vaccine during storage, transport, and after administration, ensuring that the mRNA remains intact and functional throughout the vaccine’s lifecycle (Arno et al., 2020; Sebastiani et al., 2021). One prominent example of PyMOL’s application in LNP design is the study by Zhang et al. (2023a), which explored LNP formulations for mRNA vaccines. In their work, the researchers used PyMOL to visualize and model the interaction between the lipid components of the LNPs and the encapsulated mRNA (Zhang et al., 2023a). By adjusting lipid compositions and evaluating the resulting structural and functional properties, the team optimized the LNPs to enhance mRNA encapsulation and improve delivery efficiency. The use of PyMOL in this study enabled the team to refine the nanoparticle design, resulting in a more stable and effective LNP for mRNA delivery (Arévalo-Romero et al., 2024).

### 8.2 mRNA-LNPs optimization

#### 8.2.1 Design of Experiments

Design of Experiments (DoE) is a systematic approach that allows for strategic compromises on information to significantly reduce the time and resources needed to understand and optimize a given process (Rampado and Peer, 2023), The design space is defined by the mathematical relationship between Critical Process Parameters (CPPs) and Material Attributes (CMAs) and Critical Quality Attributes (CQAs) (Politis et al., 2017). This method has been employed to optimize the formulation of mRNA-LNP vaccines by addressing critical parameters such as lipid composition, release kinetics, lipid-to-mRNA ratio, particle size, and surface charge (Tavares Luiz et al., 2021; Gurba-Bryśkiewicz et al., 2023). For cancer immunotherapy, optimizing these parameters is crucial to ensure that LNPs effectively deliver mRNA to tumor cells, improve half-life, bioavailability, and biodistribution, enhance antigen presentation, and elicit a strong immune response, for example, mRNA vaccines against COVID-19 are designed using DoE iterations to minimize or increase cell activation and to meet CQA characteristics while improving protein expression (Ly et al., 2022; Morris and Kopetz, 2022). This method also aids in fine-tuning the physicochemical properties of LNPs, including hydrodynamic diameter, zeta potential, and lipid bilayer integrity, to achieve the most effective therapeutic outcomes.

#### 8.2.2 COMSOL

COMSOL Multiphysics is a sophisticated simulation tool used to model the physical behavior of mRNA-LNP formulations (Towne et al., 2021; Zhang et al., 2024). It allows for the simulation of various nanoparticle dynamics, such as diffusion rates, aggregation behavior, and interactions with cellular membranes, and through experimental verification, COMSOL can be used to synthesize nanoparticles down to the nm level (Erdem et al., 2023). The tool supports the optimization of key parameters, including nanoparticle size distribution, release kinetics, and LNP stability under different environmental conditions.

At the same time, COMSOL allows equations from different physics domains to be solved simultaneously in the same model, allowing for a more realistic simulation of the interactions between various physics under real-world operating conditions. This approach includes modeling the behavior of LNPs in various biological fluids, their ability to traverse cellular membranes, and their release profiles under different physiological conditions. For instance, Chenguo Yao et al. team developed a dynamic electroporation model of irregular cells using COMSOL to investigate the effects of ns+mμs pulses on these cells (Yao et al., 2020). The findings by Erdem et al. regarding COMSOL simulations for optimizing flow rates and mixing efficiency in micro-reactors can be adapted to mRNA-LNP cancer therapy development. By employing COMSOL, key processes such as nanoparticle formation, encapsulation efficiency, and controlled mixing of lipids and mRNA can be simulated to ensure consistency and precision. This could optimize LNP size (e.g., 50-100 nm), improve payload stability, and refine production conditions, facilitating scalable and efficient mRNA delivery systems for enhanced therapeutic efficacy in cancer immunotherapy (Erdem et al., 2023). In a parallel effort, Li et al. leveraged advanced combinatorial chemistry and Ml to identify ionizable lipids for mRNA delivery. Ml rapidly optimized lipid libraries, improving encapsulation efficiency and targeting. COMSOL’s potential lies in enhancing such designs by simulating factors like nanoparticle stability, bioavailability, and interaction dynamics to refine LNP formulations for applications like mRNA-based cancer immunotherapy (Li et al., 2024).

## 9 AI and machine learning tools

### 9.1 XGBoost/Bayesian

XGBoost is a highly effective machine-learning algorithm known for its speed and predictive power, especially when working with structured datasets that include mRNA-LNP formulation parameters. The model employs an ensemble method that builds multiple decision trees sequentially to correct for errors made by previous trees, which allows it to handle a broad range of input variables such as lipid composition, nanoparticle size, and encapsulation efficiency. XGBoost can be optimized through hyperparameter tuning, allowing the model to refine predictions of key formulation characteristics like mRNA stability, LNP encapsulation efficiency, and delivery performance (Maharjan et al., 2024).

When combined with Bayesian optimization, this approach takes advantage of a probabilistic model to efficiently navigate the hyperparameter space. Bayesian optimization uses prior knowledge (based on previous experimental data or expert knowledge) to predict the most likely optimal formulation parameters and iteratively refines the search based on observed outcomes (Hoseini et al., 2023). For example, in mRNA-LNP optimization, Bayesian methods can help fine-tune lipid-to-mRNA ratios, lipid types (such as ionizable lipids), and nanoparticle characteristics (like surface charge or size) to maximize mRNA encapsulation, stability, and cell delivery (Sato et al., 2024). For mRNA vaccine development, XGBoost/Bayesian optimization accelerates the formulation process by systematically evaluating a range of conditions with minimal experimental trials, ensuring faster production of effective vaccine candidates. Recent applications show that combining these techniques can enhance mRNA-LNP performance, such as improving vaccine stability and optimizing lipid compositions that facilitate efficient mRNA delivery into immune cells (Castillo-Hair and Seelig, 2022). These algorithms have been proven to identify LNP formulations with the ability to trigger stronger immune responses by ensuring efficient antigen presentation and reducing the risk of immunogenicity-related adverse effects (Hoseini et al., 2024).

### 9.2 Graph Convolutional Networks

Graph Convolutional Networks (GCNs) represent a transformative application of Ml in mRNA-LNP vaccine development. These networks effectively model complex relationships inherent in graph-structured data, making them uniquely suited for tasks involving the intricate design and optimization of both mRNA sequences and their delivery systems (Gao et al., 2024). In mRNA vaccine design, GCNs encode secondary and tertiary structures of mRNA as graph networks, where nodes represent nucleotides, and edges depict structural interactions, such as hydrogen bonds or stacking interactions. This representation enables the identification of key features, like stem-loops or pseudoknots, that contribute to stability and translation efficiency, facilitating the optimization of mRNA constructs for robust antigen expression (Dorsey et al., 2024).

GCNs are also instrumental in designing LNP formulations and modeling the interactions between mRNA and lipid components, including ionizable lipids, phospholipids, and cholesterol. By treating these formulations as molecular graphs, where nodes are individual molecules and edges signify their interactions, GCNs predict encapsulation efficiency, stability during systemic circulation, and intracellular release dynamics. These predictions guide the development of formulations that enhance mRNA protection against degradation and promote efficient delivery to target cells (Wang et al., 2022). Furthermore, GCNs analyze how LNPs interact with mRNA under varying cellular conditions, such as pH fluctuations or enzymatic activity, ensuring that delivery systems are optimized for endosomal escape and subsequent mRNA release. This facilitates the effective translation of mRNA into antigens, which is crucial for activating immune cells and orchestrating adaptive immune responses. GCNs also evaluate structural variations in mRNA sequences to design antigens and immune-stimulatory adjuvants with enhanced stability and immunogenicity (Kejani et al., 2020). Through their ability to integrate complex structural, chemical, and biological data, GCNs provide a comprehensive framework for addressing challenges in mRNA-LNP vaccine development. These advancements accelerate the creation of vaccines with precise delivery mechanisms and robust immune activation, contributing to innovations in cancer immunotherapy and infectious disease prevention (Wang et al., 2024).

### 9.3 Deep Neural Networks

Deep Neural Networks (DNN) are a critical tool for mRNA-LNP immunogenicity optimization because they can model the complex, nonlinear relationships between different variables involved in vaccine development (Chen et al., 2020; Mekki-Berrada et al., 2021). DNNs consist of multiple layers of neurons that learn increasingly abstract representations of input data, which makes them highly effective in identifying intricate patterns within high-dimensional datasets. For mRNA-LNP formulation optimization, DNNs can be trained to predict how specific lipid compositions, mRNA modifications, and nanoparticle characteristics influence immune responses, such as the activation of DCs, T-cells, and B-cells (Mekki-Berrada et al., 2021; Konstantopoulos et al., 2022).

To enhance vaccine efficacy, DNNs work by analyzing large datasets that include both formulation details (lipid ratios, LNP size, and composition) and immunological endpoints (antibody production, cytokine levels, T-cell activation). The neural network identifies the relationships between formulation characteristics and immune activation, providing researchers with precise recommendations for optimizing LNP formulations that yield the strongest immune response. This can include optimizing the lipid mixture for better cellular uptake, adjusting particle size to improve lymph node targeting, or modifying mRNA constructs to enhance antigen presentation (Zeng et al., 2024).

Recent advancements in DNNs, particularly with frameworks like TensorFlow and PyTorch, have allowed the integration of mRNA-LNP optimization with high-dimensional data sources, including cellular response profiles and *in vivo* animal data (Taylor and Kriegeskorte, 2023). These networks can predict how formulation changes will affect immune responses without the need for extensive trial-and-error testing, which significantly speeds up vaccine development. In immuno-oncology, DNNs can also predict LNP formulations that target cancer cells more efficiently by analyzing tumor microenvironment data, ensuring that the mRNA vaccines not only deliver genetic material but also elicit strong, targeted immune responses against tumor cells (Chen et al., 2020). For example, DNNs can optimize formulations for enhancing the presentation of cancer antigens to immune cells in the tumor microenvironment, significantly improving the therapeutic efficacy of mRNA-based cancer vaccines (Raza et al., 2023). Additionally, DNNs can fine-tune LNP formulations to induce specific immune pathways, such as the activation of Th1 responses, which are crucial for effective anti-cancer immunity. This predictive capability is essential for designing personalized mRNA vaccines, where DNNs can assist in tailoring the vaccine to an individual’s unique immune profile, making these tools indispensable for both infectious disease and cancer immunotherapy (Jozwik et al., 2017; Castillo-Hair and Seelig, 2022).

### 9.4 Prospects

**Figure 5:**
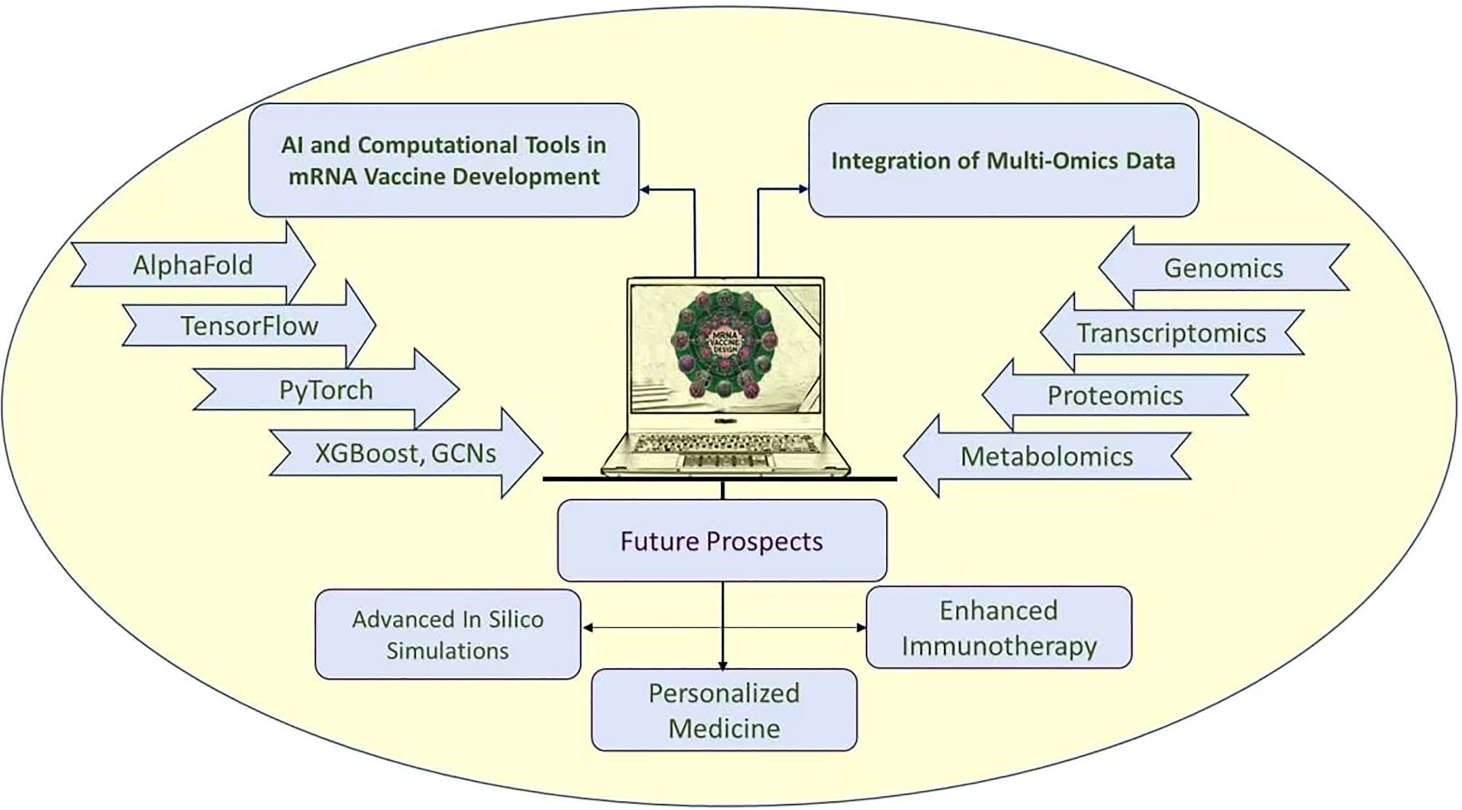
Future directions in mRNA vaccine development with AI and computational tools. This figure illustrates how AlphaFold’s accurate protein structure predictions assist in designing mRNA sequences that encode tumor-associated antigens (TAAs) and neoantigens. TensorFlow’s deep learning algorithms are used to model lipid nanoparticle (LNP) formulations, optimizing mRNA stability and delivery. PyTorch’s dynamic computational models simulate the interactions between mRNA, LNPs, and immune cells to enhance vaccine efficacy. Advanced machine learning methods, such as XGBoost, graph convolutional networks (GCNs), and deep neural networks (DNNs), are applied to refine mRNA-LNP formulations by improving mRNA stability, translation, lipid and matrix design, and immunogenicity. The integration of genomic, transcriptomic, proteomic, and metabolomic data through cutting-edge in silico simulations holds the potential to transform vaccine development, paving the way for personalized medicine and advancing cancer immunotherapy.

Undoubtedly, the future of mRNA vaccine development is advancing rapidly with the aid of advanced bioinformatics and AI tools. AlphaFold, developed by DeepMind, marks a significant advancement in predicting protein structures with high precision (Service, 2023). **Figure 5** highlights key advancements in mRNA vaccine development through the integration of bioinformatics and AI tools. The figure underscores the transformative potential of these technologies in enhancing vaccine efficacy in TME and advancing personalized immunotherapy. By utilizing deep learning techniques such as CNNs and attention mechanisms, AlphaFold provides crucial insights into how proteins fold and assemble. This capability is vital for designing mRNA vaccines that encode TAAs and neoantigens, as it allows researchers to anticipate how these proteins will behave and interact within the body. TensorFlow and PyTorch play pivotal roles in advancing mRNA-LNP vaccine technology (Xu et al., 2023). TensorFlow utilizes advanced algorithms, such as CNNs and RNNs, to model the impact of different lipid formulations on mRNA stability and delivery. This capability is crucial for optimizing LNP designs, and ensuring effective mRNA delivery to DCs, macrophages, and CTLs. PyTorch, known for its dynamic computational graph, facilitates the creation of sophisticated models to simulate interactions between mRNA, LNPs, and immune cells (Zhou et al., 2020). This flexibility is instrumental in optimizing vaccine efficacy and enhancing immune responses.

Looking ahead, the fusion of in silico simulations with multi-omics data is poised to revolutionize vaccine development. AI-driven analysis of genomic (Kamimoto et al., 2023), transcriptomic, proteomic, and metabolomic data will enable the identification of key biomarkers and pathways, leading to more targeted and effective vaccine strategies. These advancements will address current challenges in optimizing LNP formulations and mRNA stability, improving both pharmacokinetics and pharmacodynamics. Ultimately, the integration of these cutting-edge technologies holds the promise of transforming vaccine development and expanding the potential of mRNA-based therapies in personalized medicine and beyond.

## 10 Conclusion

The integration of computational biology, bioinformatics, and artificial intelligence is transforming mRNA vaccine development, enhancing their precision and effectiveness. By combining these advanced tools with machine learning, we gain deeper insights into protein structures and optimize LNP formulations and their interactions with immune cells. Additionally, incorporating MD simulations further improves our understanding of mRNA-LNPs’ structure and their interactions with cellular machinery, providing critical insights into optimizing stability and translation efficiency. These *in silico* technologies are driving progress in personalized cancer immunotherapy and opening new avenues for addressing global health challenges with next-generation vaccines.
