# Supplementary material for: Computational biology and artificial intelligence in mRNA vaccine design for cancer immunotherapy
Source: Front Cell Infect Microbiol. 2025 Jan 20;14:1501010. doi: 10.3389/fcimb.2024.1501010 (PMC11788159; doi:10.3389/fcimb.2024.1501010)
Supplement: Supplementary file 1 [file DataSheet1.docx]

**Supplement file S1**

Detailed Fluorescence Measurement and Bioinformatics of the sequencing:

**Fluorescence Intensity Calculation**: The fluorescence intensity INI_NIN​ for nucleotide NNN in Illumina sequencing is calculated using:

$$I_{N}=K. C_{N}$$

**Burrows-Wheeler Transform (BWT)**:

$$BWT\left( S \right)=last colum of sorted cyclic premutations of S$$

***FM-Index****: The FM-index is used for indexing and searching in compressed data, with:*

*where rank counts occurrences of character ccc up to position iii and select locates the iii-th occurrence.*

Bowtie employs the FM-index, described as:

$$FM-index=rank\left( c,i \right) and select (c,i)$$

where rank (c,i) counts occurrences of character (c) up to position (i), and select (c,i) gives the position of the i-th occurrence.

**FASTQC**: Base quality scores are calculated using:

$$Q= -10.\log_{10} (Pe)$$

ensuring the reliability of sequencing data.

**Trimmomatic**: This tool trims low-quality bases and adapter sequences to enhance alignment accuracy and variant detection.

**Minimap2**: Minimap2 uses the following indexing strategy for long-read alignment:

$$Minimap2=align (R,G)$$

where R is the long read and G is the reference genome. The algorithm incorporates heuristics and indexing strategies to achieve high accuracy.
